# Delayed Sternal Closure After Pediatric Cardiac Operations; Single Center Experience: a Retrospective Study

**DOI:** 10.1186/1749-8090-7-102

**Published:** 2012-10-02

**Authors:** Emre Özker, Bülent Saritaş, Can Vuran, Uygar Yörüker, Halim Ulugöl, Riza Türköz

**Affiliations:** 1 Baskent University, Istanbul medical trainning and research hospital, department of Cardiovascular Surgery, Istanbul, Turkey; 2 Baskent University, Istanbul medical trainning and research hospital, department of Anesthesiology, Istanbul, Turkey; 3 Acibadem mh.ibrahimaga konutlari, a5 d12, Kadiköy, Istanbul, Turkey

**Keywords:** Delayed sternal closure, Mediastinitis, Open heart surgery, Pediatric

## Abstract

**Background:**

Delayed sternal closure (DSC) after cardiac surgery is a therapeutic option in the treatment of the severely impaired heart in pediatric cardiac surgery. The results with the technique of DSC over a 4-year period are examined with regard to mortality and morbidity.

**Methods:**

We retrospectively reviewed records of 38 patients who had undergone DSC among 1100 congenital cardiac operations. Indication of DSC, time to sternal closure, pre and post closure cardiopulmonary and metabolic status, mortality, rate of wound and bloodstream infections were recorded.

**Results:**

The mean sternal closure time was 2.9 days. The mortality rate was 34.2% (n = 13). Twenty (52.6%) patients required prolonged antibiotic use due to postoperative infection. There was gram negative microorganism predominance. There were 4 (10.5%) patients with postoperative mediastinitis. Postoperative infection rate statistically increased with cardiopulmonary bypass time (CPBT), sternal closure time (SCT) and intensive care unit (ICU) stay time (p = 0.039;p = 0.01;p = 0.012). On the other hand, the mortality rate significantly increased with increased cross clamp time (CCT), SCT, and extracorporeal membrane oxygenation (ECMO) use (p = 0.017; p = 0.026; p = 0.03). Single ventricular physiology was found to be risk factor for mortality in delayed sternal closure (p < 0.007).

**Conclusions:**

Elective DSC does not reduce the morbidity. The prolonged sternal closure time is associated with increased rate of postoperative infection rate; therefore early closure is strongly advocated.

## Background

Open chest management and delayed sternal closure (DSC) after cardiac surgery is a therapeutic option in the treatment of the severely impaired heart especially in pediatric cardiac surgery. In the early days of cardiac surgery, primary closure of the sternum at the end of the operation was mandatory because of the concern of mediastinal infection. DSC is now accepted as a therapeutic tool in the management of hemodynamically unstable patients where cardiac compression by sternal closure is not tolerated.

Sternal closure has been shown to result in a significant decrease in cardiac output and diastolic filling, despite preserved velocity of fiber shortening, even in patients with good cardiac performance [1]. This procedure, with optimal inotropic and ventilatory assistance, can provide the necessary interim support vital for ultimate survival.

The application of the technique varies among different centers to the degree that while a few centers delay the sternal closure routinely either in all neonates or for certain pathologies, whereas others do not apply the technique. We present our results with the technique of DSC over a 4-year period to examine the outcome with special regard to mortality and morbidity.

## Methods

We retrospectively reviewed records of 1100 congenital cardiac operations performed between July 2007 and September 2011. 1011 operations were performed with median sternotomy. 38 (3.45%) patients who had undergone DSC were enrolled in the study. DSC was used after elective median sternotomy in all of the patients. The patients’ cardiac pathologies are listed in Table 1.

**Table 1:** The diagnosis and distribution of the exitus

**Diagnosis**	**Number of patients (n = 38)**	**Number of Deceased Patients (n = 13)**
HLHS	7	7
TGA	9	-
TGA + VSD	10	-
CAVSD	1	-
CAVSD + TA	1	1
TAPVC	4	2
TOF	1	1
DORV	1	1
AO INTER	1	-
VSD	3	1

Table 1 demonstrating the cardiac pathologies of the patients. (HLHS: Hypoplastic Left Heart Syndrome; TGA: Transposition of Great Arteries; VSD: Ventricular Septal Defect; CAVSD: Complete Atrioventricular Septal Defect;TA: Tricuspid Atresia; TAPVC: Total Anomalous Pulmonary Vein Connection; TOF: Tetralogy Of Fallot; DORV: Double Outlet Right Ventricle; AO INTER: Aortic Interruption).

Indication of DSC, time to sternal closure, pre and post closure cardiopulmonary and metabolic status including mean heart rate, systolic blood tension, left atrial pressure, lactate level and inotrope score, ventilator support duration, mean intensive care unit (ICU) and mean hospital stay times were recorded. Inotrope score was calculated such that dopamine, dobutamine were assigned a score of 1 per μg/kg/min administered, milrinone and adrenaline was assigned scores of 10 and 100 μg/kg/min, respectively. A patient receiving dopamine at 5 μg/kg/min concurrently with milrinone at 0.5 μg/kg/min and adrenaline at 0.1 μg/kg/min was calculated to have an inotrope score of 20 ([1x5] + [10x0.5]+ [100x0.1]). Pediatric ICU mortality, wound infections, and bloodstream infections were recorded. The patients who had been treated for sterile wound infection (SWI), deep sternal wound infection (DSWI) and other infections were noted. SWI was defined as inflammatory changes and non-purulent drainage on the sternal incision confirmed with clear cultures. DSWI was defined as the presence of pus in the retrosternal space and isolation of organisms from retrosternal cultures obtained at revision operations or debridement. The patients with SWI, DSWI and other infections along with the organisms detected from cultures were summarized in Table 2. Delayed sternal closure (DSC) is defined as delaying the sternal closure either as a principal method or after failure of one or several trials of closure at the end of the operation. The main reason for leaving the sterna open at the end of the procedures was low cardiac output.

**Table 2:** Postoperative infection data

**Patient no**	**Diagnosis**	**Site of infection**	**Organism detected**	**Result**
1.	VSD	Blood	Staf A	EX
2.	HLHS	Blood	Klebs P	EX(LCO)
3.	HLHS	Blood	Staf A	EX
4.	HLHS	Blood	Enterococ A,Staf A	EX
5.	TOF	Blood	Klebs P,Staf A	EX
6.	HLHS	Blood	VRE, Candida A	EX (LCO)
7.	TAPVC	Blood	Acinetobac	EX
8.	TAPVC	Blood	Staf A,Candida A	EX
9.	HLHS	Blood	Candida A, Acinetobac,VRE	EX
10.	TGA	Sputum	Candida A	
11.	TGA	Blood	VRE	
12.	TGA	Sternum(SWI)	SWI	
13.	TGA	Sternum(SWI)	SWI	
14.	CAVSD	Sputum	Acinetobac	
15.	TGA + VSD	Sternum(SWI)	SWI	
16.	TGA + VSD	Sternum	Enterobact A	
17.	TGA	Blood/Sternum (SWI)	Candida A	
18.	TGA	Sputum	Acinetobac	
19.	TGA	Blood/Urinary inf	Staf A/Enterobact A	
20.	VSD	Sternum(SWI)	SWI	
21.	TGA + VSD	Sternum	Klebs P	
22.	AO INTER	Blood	Klebs P	
23.	TGA + VSD	Sternum	Enterobact A	
24.	TGA	Blood	Klebs P,Pseudomonas A	
25.	TGA	Urinary inf/Sternum(SWI)	Klebs P	
26.	TAPVC	Blood/Sputum	Staf A/Pseudomonas A	
27.	TGA + VSD	Sputum	Pseudomonas A	
28.	TGA + VSD	Blood	Enterobact A	
30.	TGA + VSD	Sternum	Staf A	

Table 2 showing site of infection and the detected organisms with the clinical outcome after treatment. (Urinary inf: Urinary infection; Staf A: Stafilococcus Aureus; Klebs P: Klebsiella Pneumonia; Enterococ A: Enterococcus Aeruginosa; VRE: Vancomycin resistant Enterococcus; Acinetobac: Acinetobacter; Enterobact A: Enterobacter Aerogenes; Candida A: Candida Albicans; SWI: Sterile wound infection; Pseudomonas A: Pseudomonas Aeruginosa;EX: Exitus); LCO: Low cardiac output.

All patients were operated on had a cardiopulmonary bypass (CPB) with or without aortic cross-clamping. The CPB was performed with a basic flow of 2.5 l/min per m^2^. Myocardial preservation was performed by anterograde blood cardioplegic solution. We used normothermic blood cardioplegia with warm induction and reperfusion. The average interval between the cardioplegia doses was about 15 min. The ultrafiltration was used during CPB and after CPB (modified ultrafiltration) in all cases.

Following the termination of cardiopulmonary bypass, the patients with unstable hemodynamics (low blood tension, rhythm disturbances, high left atrial pressure) were left with chests open for 45–60 minutes until the hemodynamic parameters recovered. In these patients sterna were left open:

1. If high inotropic support were needed (>5 μg/kg/min dopamine, >0.5 μg/kg/min milrinone and >0.2 μg/kg/min adrenaline),

2. If the blood lactate level was above 5 mmol/L in the arterial blood gas and showed incline in consecutive measurements,

3. If mean and systolic blood pressures were less than 45 and 55 mmHg, respectively.

In case of failure of the trial using the chest retractor, a rigid plastic material (we usually use the plastic holder of the sutures and trim them according to the size of the sternum) was fixed in place to keep the chest widely opened and an airtight synthetic transparent patch, usually 2 layers of nylon serum bag, was used to cover the sternal gap which is sutured to the skin with running polypropylene sutures. The dressing was changed in ICU in a totally aseptic manner every day. In some cases sterna were left open but skins were closed primarily. The patient is evaluated for sternal closure on daily basis. If a stable hemodynamic condition; a negative total fluid balance and an improvement of the respiratory dynamics and arterial blood gases; and decrease in blood lactate level in the last 24 hours were maintained, then the patient was taken to OR for closure trial. The trial of closure was identical to the intraoperative trial. Before each trial, two mediastinal swabs for culture were taken.

In the case of a successful trial, the sternum was closed with steel wires and the deep subcutaneous tissue also with absorbable suture. The skin was then closed with separated interrupted vertical mattress sutures. However recently we start to delay sternal closure 1 to 2 hours during the initial operation in cases with limited hemodynamics. In the interim we administer diuretics and apply lower tidal volume. The number of cases in whom we achieved to close the sterna primarily with this manipulation has increased lately.

Our routine antibiotic prophylaxis was cephazoline 50 mg/kg at the induction of anesthesia and 50 mg/kg on the bypass, then switching to vancomycin 40–60 mg/kg/day and imipenem 100–150 mg/kg/day, the dosages were adjusted according to the renal functions in case of DSC until the removal of drains. The antibiotic regimen was then changed according to the cultures.

### Statistical analysis

The data were compared with a two-tailed paired *t*-test. Comparisons between groups of unequal populations were achieved with use of a two-tailed unpaired *t*-test assuming unequal variances or the Wilcoxon rank sum test, or with both tests. Univariate analysis and multivariate logistical regression were used to determine predictors for DSC. A value of p < 0.05 was considered significant. Analysis was performed using SPSS 16.0 (SPSS Inc, Chicago, IL).

## Results

There were 38 patients who were enrolled in the study. 15 (39.5%) patients were female and 21 (55.3%) patients were male. The mean age was 38.5 ± 85 days (4 to 510). The mean body weight was 3488.2 ± 940 grams (2100 to 7700). There were no reoperations and no history of previous sternotomy. There was only one patient with preoperative infection. The mean ASA score was 3.4 ± 0.48 (14 patients had ASA score of 4). The mean cross clamp time (CCT) was 77.7 ± 31.8 minutes (27 to 174) and the mean cardiopulmonary bypass time (CPBT) was 180.9 ± 62.3 minutes (20 to 309). 5 (13.2%) patients had ECMO in the postoperative term. 27 (71.1%) patients were weaned from ventilator support. The mean duration of ventilation was 16.7 ± 33 days (3 to 201). The mean length of stay in the intensive care unit was 26.8 ± 40.3 days (4 to 208) and the mean hospital stay time was 36,7 ± 43.8 days (4 to 245). The mean sternal closure time was 2.9 ± 2.3 days (16 hours to 10 days). The mean follow up time was 153.2 ± 194.7 days (15 to 1020 days). The mortality rate was 34.2% (n = 13).

Twenty (52.6%) patients required prolonged antibiotic use due to postoperative infection. The most common microorganism detected was Enterobacter aerogenes (20%) and Klebsiella Pneumonia (20%). Gram negative microorganisms were detected more than gram positive organisms (13 patient vs 3 patients). Six (15.8%) children had minor wound dehiscence and sterile mediastinal discharge (sterile wound infection), which subsequently healed by secondary intention, however there were 4 (10.5%) patients with postoperative mediastinitis with positive wound swabs requiring surgical reexploration and antibiotic treatment. There was gram negative predominancy in the sternal cultures (Table 2). Thirteen (34.25%) patients died during the treatment. Three (7.9%) among these 13 patients couldn’t have been reoperated for sternal closure. The mortality was related to sepsis in 7 (18.4%), low cardiac output syndrome in 5 (13.2%) and to sudden cardiac death in 1 (2.6%) patient.

Sternum closure time (SCT) was significantly longer in patients with longer cardiopulmonary bypass time (CPBT) (p = 0.043). The relationship between SCT and lactate level before sternal closure was significant (p = 0.031). Low patient weight was significantly related to longer cross clamp time (CCT)(p = 0.036). The lactate level after sternal closure was significantly higher than the lactate level before closure (mean 2.73 ± 2.8 and 8.16 ± 1.3 mmol/L, respectively;(p = 0.01). The blood tension significantly increased after sternal closure (mean blood tension before and after closure: 66.3 ± 11.3 and 76.4 ± 14.9 mmHg, respectively (p = 0.001) and the inotrope score significantly decreased after closure (mean inotrope score before and after closure: 34.6 ±21.5 and 25.2 ± 14.8, respectively (p = 0,0). However the difference between heart rate and left atrial pressures determined before and after sternal closure was statistically insignificant.

Postoperative infection rate statistically increased with CPBT, SCT and ICU stay time (p = 0.039;p = 0.01;p = 0.012). The relationship between mediastinitis and patient age, patient weight, patient gender, CPBT, CCT, ICU stay time and ECMO use were statistically insignificant. There was statistically strong relationship between longer SCT and increased rate of mediastinitis (p = 0.015). The total number of mediastinitis cases was 12 (1.09%) where 4 cases were among 38 DSC cases (10.5%). The mediastinitis rate was 0.72% when the DSC group was excluded. The difference between the rates of mediastinitis in two different techniques is statistically significant (p = 0.0008).

There was no significant relationship between mortality and patient age, patient weight, CPBT, ICU stay time. On the other hand, the mortality rate significantly increased with increased CCT, SCT, and ECMO use (p = 0.017; p = 0.026; p = 0.03). Single ventricular physiology was found to be risk factor for mortality in delayed sternal closure (p < 0.007).

## Discussion

Open sternotomy and DSC is a surgical technique that has been used in children to facilitate postoperative recovery. Cardiac compression, which may occur at the time of sternal closure, can lead to decreased cardiac output and hemodynamic instability. Riahi and colleagues were the first to point out the problem of postoperative cardiomediastinal disproportion in 1975 [2]. Surgical manipulation of the heart leading to swelling in the pericardium and/or the pericardiomediastinal space, bleeding at the end of CPB, significant increase in heart size with severe ventricular dysfunction, reperfusion related myocardial edema and relentless arrhythmias are all severe complications of cardiac operations that preclude the sternum to be closed [3-5]. They are often associated with a prolonged perfusion time and poor myocardial preservation [3]. Treatment by DSC provides time for recovery of myocardium and for treating the bleeding complications.

The potential for benefit from DSC is greater in small children, because of a larger cardiac size relative to the thoracic cavity. The utility of DSC in children has become evident over the last two decades. While shifting from palliative to earlier corrective surgery, procedures with longer CPB times on younger patients and DSC have become more common. Furnary and colleagues have recently demonstrated after re-openning the sternal incision, up to 59% increase in cardiac index and 18% rise in systemic blood pressure, without significant change in cardiac filling pressures could be obtained [3]. It has been suggested that DSC can be used to prevent the development of low cardiac output state [6]. Some of the pediatric cardiac surgery programs currently use an “elective sternal opening from the operating room” strategy for patients with marginal hemodynamic profiles. In many pediatric institutions it is routine to leave the sternum open prophylactically following long operations or specific procedures such as Norwood I for hypoplastic left heart syndrome [7].

The optimal time to sternal closure remains unclear. In all cases the decision is being made usually by personally based criteria which are highly subjective. There are surgeons who generally leave the sternum open for at least 3 days, and close it any time from 2 to 14 days postoperatively [7,8]. There are also others who have aggressive approach to DSC, aiming for closure within 24 hours [9]. We don’t have strict criteria for the timing of the sternal closure. We don’t want to prolong the closure time over 3 days for infection purposes; however we usually close the chest when the patient becomes hemodynamically stable. In fact SCT prolonged up to 10 days in one of our patient who was then died to septicemia. The mean SCT in our study was 2.97 days.

Many techniques for the maintenance of an open sternum have been described in the literature including direct closure of the skin, or adaptation of the skin with a latex membrane (Esmark Bandage) sewn to the skin edges or VAC treatment [10].We prefer a transparent nylon serum bag. It is easy to handle, cheap and helps monitor the bleeding and clot evacuation to avoid tamponade.

Potential risks of delayed sternal closure include sepsis, mediastinitis, bleeding, and late sternal instability. Infectious complications are already well-known contributors to postoperative morbidity and mortality after pediatric open heart surgery [8]. Kagan and colleagues demonstrated that an American Society of Anesthesiologists score of 4 or greater was risk factor for development of mediastinitis [11]. Tabbutt found mortality as 19%, surgical site infection as 6.7% and mediastinitis as 3.9% in his study of delayed sternal closure in pediatric patients. [8]. Shin reported postoperative infection and sterile wound dehiscence rate in DSC patients as 11% and 9%, respectively [12]. These sternal wound infections have been associated with longer postoperative stay [8,13-15]. The group of patients undergoing DSC are believed to have an increased risk for infection because they have predisposing factors such as: prolonged CPB time, low cardiac output, massive transfusion and the need for multiple re-explorations of the chest. A review of the literature reveals that the incidence of wound infection after DSC is generally less than 10% [8]. Some have reported that DSC is not associated with an increase in surgical site infections [6,8,14,16-19], besides some authors claim that wound complications are reduced in DSC technique [20]. Others report DSC as a significant risk factor for bloodstream and surgical site infections [8,13]. Das reported a fourfold increased risk of developing bloodstream infection in the DSC group.

In Woodwards’ study determining the rate of mediastinitis among the pediatric cardiac centers, 38 (43%) of the 89 centers were enrolled in the study. Woodwards reported 8,774 pediatric congenital procedures with a mean mediastinitis rate of 1.53%. The study concluded that DSC was not associated with increased incidence of mediastinitis. Variations in preoperative measures, antibiotic regimens, and wound care among these centers didn’t statistically influence the incidence of mediastinitis [21]. Between July 2007 and September 2011, 1100 pediatric cardiac operations were performed in our center. The total number of mediastinitis cases was 12 (1.09%) in our cohort in which 4 cases initially had undergone delayed sternal closure [4 out of 38 DSC cases (10.5%)]. The difference between the rates of mediastinitis in two different techniques is statistically significant. Unlike Woodwards and colleagues, we detected strong correlation between DSC and mediastinitis in our study.

It should be kept in mind that most studies of DSC, including ours, have evaluated children of all age groups along with the younger infants who may well be at a higher risk of septic complications. When the mean ages of mediastinitis cases in our center were compared, the mean age in DSC group is less than the mean age of the rest of the mediastinitis cases (23 to 342.5 days); however the difference was not found to be statistically meaningful due to high standard deviation (p = 0,248). However, age was not shown to be risk factor for mediastinitis in this study, instead SCT was associated with increased rate of mediastinitis. CPBT,CCT and ICU stay time were not related to mediastinitis risk.

Prolonged SCT was also shown to be a risk factor for postoperative infection. The infants with open chest are potentially critically ill for a variety of reasons that led to DSC. This may itself be a factor contributing to prolonged ICU stay which we also showed to be strongly related with high rate of postoperative infections in our study. The most common microorganism we isolated from the cultures was gram negative which is in agreement with the literature [8,13,21].

Our study showed that longer durations of CPB and cross clamp was associated with longer SCT and high lactate levels before closure indicating the negative compromise of CPB on hemodynamics. With respect to changes in key physiologic parameters over time, we found that although mean heart rate and left atrial pressures were unchanged, mean blood pressure increased and inotrope score decreased in 12 hours after sternal closure which was in contradiction with Tabbutt and Horvath’s studies suggesting decrease in hemodynamic performance after sternal closure [8,22].

## Conclusion

Use of DSC is an important management strategy for congenital cardiac surgery, particularly for very young infants who undergo meticulous and long cardiac procedures. Elective DSC does not reduce the morbidity but it confirms the safety and efficacy of the cardiac procedure. However, DSC in single ventricle physiology was shown to be associated with increased mortality. Instead of delayed sternal closure, ECMO or assist device may be preferred in this group of patients. The prolonged sternal closure time is associated with increased rate of postoperative infection rate; therefore early closure is strongly advocated. However we believe it is crucial to demonstrate hemodynamic stability and decide for the optimal timing on case-by-case decision.

## Competing interests

The authors declare that they have no competing interests.

## Authors’ contributions

EÖ designed the study, collected the data and wrote the manuscript. BS collected the data and participated in the clinical work. CV worked as a co-surgeon in the clinic. UY worked as a co-surgeon in the clinic. HU worked as the anesthesiologist and contributed in writing the study. RT worked as the chief surgeon and contributed in supervising and critical reviewing the manuscript. All authors read and approved the final manuscript.

## References

[B1] Gielchinsky I Parsonnet V Krishnan B Silidker M Abel RM Delayed sternal closure following open-heart operation Ann Thorac Surg 1981 32 273 277 10.1016/S0003-4975(10)61050-8 7025771

[B2] Riahi M Tomatis LA Schlosser RJ Bertolozzi E Johnston DW Cardiac compression due to closure of the median sternotomy in open-heart surgery Chest 1975 67 113 114 10.1378/chest.67.1.113 1235315

[B3] Furnary AP Magovern JA Simpson KA Magovern GJ Prolonged open sternotomy and delayed sternal closure after cardiac operations Ann Thorac Surg 1992 54 233 239 10.1016/0003-4975(92)91375-J 1637210

[B4] Pye S McDonnell M Nursing considerations for children undergoing delayed sternal closure after surgery for congenital heart disease Crit Care Nurse 2010 30 50 61 10.4037/ccn2010712 20515883

[B5] Yasa H Lafcı BB Yılık L Bademci M Sahin A Kestelli M Yeşil M Gürbüz A Delayed sternal closure: an effective procedure for life-saving in open-heart surgery Anadolu Kardiyol Derg 2010 10 163 167 10.5152/akd.2010.043 20382617

[B6] Alexi-Meskishvili V Weng Y Uhlemann F Lange PE Hetzer R Prolonged open sternotomy after pediatric open heart operation: experience with 113 patients Ann Thorac Surg 1995 59 379 383 10.1016/0003-4975(94)00840-4 7847952

[B7] Long CB Shah SS Lautenbach E Coffin SE Tabbutt S Gaynor JW Bell LM Postoperative mediastinitis in children: epidemiology, microbiology, and risk factors for gram negative pathogens Pediatr Infect Dis J 2005 24 4 315 319 10.1097/01.inf.0000157205.31624.ed 15818290

[B8] Tabbutt S Duncan BW, McLaughlin D, Wessel DL, JonasRA Laussen PC. Delayed sternal closure after cardiac operations in a pediatric population. J Thorac Cardiovasc Surg 1997 113 886 893 10.1016/S0022-5223(97)70261-7 9159622

[B9] Estrera AL Porat EE Miller CC 3rd Meada R Achouh PE Irani AD Safi HJ Outcomes of delayed sternal closure after complex aortic surgery Eur J Cardiothorac Surgery 2008 33 1039 1042 10.1016/j.ejcts.2008.02.025 18359240

[B10] Fleck T Kickinger B Moidl R Waldenberger F Wolner E Grabenwoger M Wisser W Management of open chest and delayed sternal closure with the vacuum assisted closure system: preliminary experience Interact Cardiovasc Thorac Surg 2008 7 801 804 18541606 10.1510/icvts.2008.177527

[B11] Shin HJ Jhang WK Park JJ Yun TJ Impact of delayed sternal closure on postoperative infection or wound dehiscence in patients with congenital heart disease Ann Thorac Surg 2011 92 705 709 10.1016/j.athoracsur.2011.03.040 21801923

[B12] Pollock EM Ford-Jones EL Rebeyka I Mindorff CM Bohn DJ Edmonds JF Lightfoot NE Coles J Williams WG Trusler GA Early nosocomial infections in pediatric cardiovascular surgery patients Crit Care Med 1990 18 378 384 10.1097/00003246-199004000-00006 2318048

[B13] Elella RA Najm HK Balkhy H Bullard L Kabbani MS Impact of blood stream infection on the outcome of children undergoing cardiac surgery Pediatr Cardiol 2010 31 483 489 10.1007/s00246-009-9624-x 20063161

[B14] McElhinney DB Reddy VM Parry AJ Johnson L Fineman JR Hanley FL Management and outcomes of delayed sternal closure after cardiac surgery in neonates and infants Crit Care Med 2000 28 1180 1184 10.1097/00003246-200004000-00044 10809302

[B15] Johnson JN Jaggers J Li S O'Brien SM Li JS Jacobs JP Jacobs ML Welke KF Peterson ED Pasquali SK Center variation and outcomes associated with delayed sternal closure after stage 1 palliation for hypoplastic left heart syndrome J Thorac Cardiovasc Surg 2010 139 1205 1210 10.1016/j.jtcvs.2009.11.029 20167337 PMC2907662

[B16] Kagen J Lautenbach E Bilker WB Matro J Bell LM Dominguez TE Gaynor JW Shah SS Risk factors for mediastinitis following median sternotomy in children Pediatr Infect Dis J 2007 26 7 613 618 10.1097/INF.0b013e31806166bb 17596804

[B17] Mehta PA Cunningham CK Colella CB Alferis G Weiner LB Risk factors for sternal wound and other infections in pediatric cardiac surgery patients Pediatr Infect Dis J 2000 19 1000 1004 10.1097/00006454-200010000-00012 11055604

[B18] Allpress AL Rosenthal GL Goodrich KM Lupinetti FM Zerr DM Risk factors for surgical site infections after pediatric cardiovascular surgery Pediatr Infect Dis J 2004 23 231 234 10.1097/01.inf.0000114904.21616.ba 15014298

[B19] McAnally HB Cutter GR Ruttenber AJ Clarke D Todd JK Hypothermia as a risk factor for pediatric cardiothoracic surgical site infection Pediatr Infect Dis J 2001 20 459 462 10.1097/00006454-200104000-00023 11332681

[B20] Main E Elliott MJ Schindler M Stocks J Effect of delayed sternal closure after cardiac surgery on respiratory function in ventilated infants Crit Care Med 2001 29 1798 1802 10.1097/00003246-200109000-00024 11546989

[B21] Woodward CS Son M Calhoon J Michalek J Husain SA Sternal wound infections in pediatric congenital cardiac surgery: a survey of incidence and preventative practice Ann Thorac Surg 2011 91 799 804 10.1016/j.athoracsur.2010.10.030 21353002

[B22] Horvath R Shore S Schultz SE Rosenkranz ER Cousins M Ricci M Cerebral and somatic oxygen saturation decrease after delayed sternal closure in children after cardiac surgery J Thorac Cardiovasc Surg 2010 139 894 900 10.1016/j.jtcvs.2009.06.013 19660343

